# HBV Core Promoter Inhibition by Tubulin Polymerization Inhibitor (SRI-32007)

**DOI:** 10.1155/2020/8844061

**Published:** 2020-10-14

**Authors:** Raj Kalkeri, Junzhong Peng, Chunsheng Huang, Zhaohui Cai, Roger G. Ptak, Mark J. Suto

**Affiliations:** ^1^Infectious Disease Research, Drug Development Division, Southern Research, Frederick, MD, USA; ^2^Drug Discovery, Southern Research, Birmingham, AL, USA

## Abstract

Approximately 257 million people chronically infected with hepatitis B virus (HBV) worldwide are at risk of developing hepatocellular carcinoma (HCC). However, despite the availability of potent nucleoside/tide inhibitors, currently there are no curative therapies for chronic HBV infections. To identify potential new antiviral molecules, a select group of compounds previously evaluated in clinical studies were tested against 12 different viruses. Amongst the compounds tested, SRI-32007 (CYT997) demonstrated antiviral activity against HBV (genotype D) in HepG2.2.2.15 cell-based virus yield assay with 50% effective concentration (EC_50_) and selectivity index (SI) of 60.1 nM and 7.2, respectively. Anti-HBV activity of SRI-32007 was further confirmed against HBV genotype B in huh7 cells with secreted HBe antigen endpoint (EC_50_ 40 nM and SI 250). To determine the stage of HBV life cycle inhibited by SRI-32007, time of addition experiment was conducted in HepG_2_-NTCP cell-based HBV infectious assay. Results indicated that SRI-32007 retained anti-HBV activity even when added 72 hours postinfection (72 h). Additional mechanism of action studies demonstrated potent inhibition of HBV core promoter activity by SRI-32007 with an EC_50_ of 40 nM and SI of >250. This study demonstrates anti-HBV activity of a repurposed compound SRI-32007 through inhibition of HBV core promoter activity. Further evaluation of SRI-32007 in HBV animal models is needed to confirm its activity in vivo. Our experiments illustrate the utility of repurposing strategy to identify novel antiviral chemical leads. HBV core promoter inhibitors such as SRI-32007 might enable the development of novel therapeutic strategies to combat HBV infections.

## 1. Introduction

Worldwide approximately 257 million people are chronically infected with hepatitis B virus (HBV) (Global Hepatitis Report, 2017 World Health Organization). Long-term infection with HBV increases the risk for clinical complications ranging from cirrhosis to liver failure and hepatocellular carcinoma (HCC). With an estimated 887,000 deaths globally, HBV constitutes an international public health problem.

HBV lifecycle (reviewed in [1]) involves entry into hepatocytes through sodium taurocholate cotransporting polypeptide (NTCP) receptor and heparin sulfate proteoglycans. After infection, HBV virions uncoat in the cytoplasm, and the viral nucleocapsids are transported into the nucleus, where a mixture of host and viral enzymes convert the partially double-stranded viral relaxed circular DNA (rcDNA) into covalently closed circular DNA (cccDNA). cccDNA is crucial for formation of subgenomic RNA (sgRNA) and pregenomic RNA (pgRNA). sgRNA produces the polymerase (*P*) protein, which gets packaged into the viral nucleocapsid along with the pgRNA. In the nucleocapsid, the reverse transcription of pgRNA leads to the production of viral negative and positive strand DNA, both of which lead to the formation of viral rcDNA. Nucleocapsids contribute to either virus formation/maturation or cccDNA amplification. In the virus formation pathway, a portion of the nucleocapsids containing the rcDNA matures via the endoplasmic reticulum (ER) and gets excreted through the cellular protein secretion pathways as “infectious viral particles.” In the cccDNA amplification pathway, nucleocapsids recycle back into the nucleus to amplify cccDNA and produce more sgRNA and pgRNA.

Current standard of care (SOC) options to treat HBV infections are limited and include either long-term therapy with nucleoside analogs (such as lamivudine, adefovir, entecavir, tenofovir, and alafenamide) or interferon (conventional as well as PEGylated IFN-*α*). However, SOC can only suppress viral replication (reviewed in [2]) but cannot eliminate HBV infection [3], due to both viral (the presence of cccDNA in the nucleus of the infected cells) and host (ineffective host immune response) factors. As HBV patients require SOC treatment throughout their life, drug toxicity and emergence of viral resistance are commonly observed adverse events. In addition to SOC, many therapeutics targeting different stages of the viral life cycle are currently in clinical testing (reviewed in [4]). These include HBV entry inhibitor (Myrcludex B), capsid assembly modifiers (AT-61, AT-130, NVR3-778, etc.), HBs antigen inhibitors (REP 2139, REP 2165), polymerase inhibitor (Besifovir), and viral RNA interference (ARC-520 and ARC-521). As drugs acting on various stages of viral life cycle could complement and synergize in their antiviral activity, there is a need for effective antivirals targeting different stages of HBV life cycle in order to eliminate the virus.

Repurposing of drugs or compounds for multiple disease indications is an emerging concept in drug discovery [5]. As many of the repurposed drugs have already been evaluated for human safety and pharmacokinetics, drug repurposing can accelerate the drug development process. To evaluate repurposed drugs/compounds for various indications, Southern Research, an institution dedicated to drug discovery, has acquired libraries of compounds, including FDA-approved drugs as well as approximately 400 compounds that have failed in clinical trials either due to lack of efficacy or safety. From the latter group, nucleoside analogs were also excluded as the focus was on traditional small molecules. Hundred (100) compounds were selected based upon their structure (drug-like properties, molecular weight, and reactive groups) and screened for antiviral activity against multiple viruses (hepatitis B virus, HSV-1, HCMV, Rift Valley fever, Venezuelan equine encephalitis virus, Tacaribe virus, hepatitis C virus, yellow fever virus, poliovirus, vaccinia virus, adenovirus-5, and norovirus) through the preclinical screening program of National Institute of Allergy and Infectious Diseases (NIAID). The primary goal of this study was to identify compounds that could be advanced expeditiously into clinical trials. The secondary goal was to identify new antiviral mechanisms as all of the compounds selected have reported mechanism of action (MOA). Out of the 100 compounds tested, two compounds demonstrated activity against the HBV virus and selectivity index to warrant further evaluation in secondary HBV assays. The compounds were lexibulin (2-pyrimidinamine, 5-[6, 7-dihydro-7-(methylsulfonyl)-2-(4-morpholinyl)-5H-pyrrolo[2, 3-d] pyrimidin-4-yl]-) and SRI-32007 (Cyr997) (5-(7-(methylsulfonyl)-2-morpholino-6, 7-dihydro-5H-pyrrolo-[2, 3-d] pyrimidin-4-yl) pyrimidin-2-amine). Both compounds were tested in the assay and only SRI-32007 confirmed its antiviral activity against HBV. SRI-32007 (CYT997), a tubulin polymerization inhibitor, is orally bioavailable in rats and mice with favorable pharmacokinetic properties [6]. We describe here the antiviral activity of SRI-32007 against HBV (genotypes B and D) using virus yield assay, secreted HBe antigen (Ag), infectious virus assay, and further demonstrate that SRI-32007 exerts its anti-HBV activity through inhibition of HBV core promoter.

## 2. Materials and Methods

### 2.1. Compounds

Lamivudine was acquired from NIH AIDS reagent repository. Myrcludex B was provided in kind from Dr. Stefan Urban, Universitatsklinikum Heidelberg, Germany. Entecavir and SR-32007 were procured from Selleckchem, Munich, Germany.

### 2.2. Antiviral Assay against HBV Genotype D in HepG2.2.2.15 Cells

The primary anti-HBV assay was performed as previously described [7, 8], with the modification of addition of real-time quantitative polymerase chain reaction (qPCR) to quantify extracellular HBV DNA copies associated with virions released from HepG2 2.2.15 cells. Briefly, HepG2 2.2.15 cells were plated in 96-well microtiter plates at 1.5 × 10^4^ cells/well in Dulbecco's Modified Eagle's Medium (DMEM) supplemented with 2% FBS, 380 *μ*g/mL G418, 2.0 mM L glutamine, 100 units/mL penicillin, 100 *μ*g/mL streptomycin, and 0.1 mM nonessential amino acids. After 16–24 hours, the confluent monolayer of HepG2 2.2.15 cells was washed, and the medium was replaced with complete medium containing various concentrations of SRI-32007 or lamivudine in triplicates. Three days later, the culture medium was replaced with fresh medium containing either SRI-32007 or lamivudine. Six days following the initial treatment with the SRI-32007 or lamivudine, the cell culture supernatants were collected and treated with pronase followed by quantitation of HBV DNA using qPCR. Antiviral activity was calculated from the reduction in HBV DNA levels (EC_50_ values). A tetrazolium dye (MTS; 3-(4, 5-dimethylthiazol-2-yl)-5-(3-carboxymethoxyphenyl)-2-(4-sulfophenyl)-2H-tetrazolium; CellTiter®96 Reagent (Promega, Madison, WI)) uptake assay was employed to measure cell viability and calculate compound cytotoxicity (CC_50_). The ratio of cell viability (CC_50_) to anti-viral activity (EC_50_) was used to calculate the selectivity index (SI_50_). Hepatitis B virus e (HBe) antigens in the SRI-32007/lamivudine-treated HepG2 2.2.15 cell culture supernatants were measured using hepatitis B virus *e* antigen (HBe Ag) AlphaLISA Detection Kit (AL3082, PerkinElmer) following the manufacturer's protocol.

### 2.3. Antiviral Assay against HBV Genotype B in Huh7 Cells

Huh7 cells were seeded in T-75 cell culture flask using Dulbecco's Modified Eagle's Medium (DMEM) supplemented with 10% FBS. Cells were transfected with 6.75 *μ*g pHBV 1.3-B6.2 (kind gift from Dr Lih-Hwa Hwang, National Yang-Ming University, Taipei, Taiwan) plasmid, using Lipofectamine 2000 reagent (ThermoFisher, Waltham, MA) according to the manufacturer's protocol. Four hours after transfection, cells were washed with PBS, trypsinized, and seeded in 96-well plate at 20,000 cells/well density, in the presence of serial dilutions of SRI-32007. Six days post-treatment, viability of the cells was measured by MTS assay as described previously for HepG2.2.2.5 cells. HBe antigen levels in the cell culture supernatant was measured by HBe antigen ELISA (Cat#WB2496, Xpressbio, Frederick, MD) using the kit protocol with slight modification. Briefly, 25 *μ*L of cell culture supernatant was transferred to each well of ELISA plate in the presence of 25 *μ*L of PBS with 50 *μ*L of HRP-conjugate and incubated for 60 minutes at 37°C. ELISA plate was washed five times with ELISA washing buffer, three times with PBS followed by addition of 100 *μ*L of QuantaRed Substrate (enhanced chemifluorescent substrate) working solution (Cat#15159, Thermo Fisher Scientific, Waltham, MA), and incubated for 15 minutes. Ten microliter of QuantaRed stop solution was added to each well, followed by shaking for 30 seconds and reading in the Spectramax I3 ELISA plate reader (Molecular Devices) (top read) at the 530 nm excitation and 585 nm emission wavelengths. HBe Ag levels in the compound-treated wells were normalized to the virus-infected wells without any compound treatment (virus control, VC). EC_50_ was calculated as the compound concentration at which the HBe antigen secretion was reduced by 50% compared to the virus control (VC).

### 2.4. HBV Infection Assay

The HBV infection assay was performed in HBV infected HepG_2_-NTCP cells as previously described [9] with ELISA based HBe antigen measurement in the infected cell culture supernatants as an endpoint. The HepG_2_-NTCP-C4 cell line is a human hepatoblastoma cell line with stable expression of bile acid transporter (NTCP). Briefly, HepG_2_-NTCP-C4 cells (Dr. Watashi, National Institute of Infectious Diseases, Japan) were plated in collagen coated 96-well microtiter plates at 2.5 × 10^4^ cells/well using DMEM/F-12 + GlutaMax (Invitrogen) supplemented with 10 mM HEPES (Invitrogen), 100 units/ml penicillin, 100 *μ*g/ml streptomycin, 10% FBS, 50 *μ*M hydrocortisone, and 5 *μ*g/ml insulin in the presence of 400 *μ*g/ml G418. After 16–24 hours, culture medium was replaced with complete medium containing 3% DMSO for 24 hours to allow hepatocyte differentiation. The following day, cells were preincubated with different dilutions of SRI-32007 or Myrcludex B for 30 minutes, followed by infection with HBV (isolated from HepG2.2.2.15 cells) in virus-infection medium (growth medium containing 4% PEG8000 and 3% DMSO) for 16 hours. Wells treated with media alone (cell control, CC) or virus alone (virus control, VC) were used as controls. Cells were washed with PBS three times after infection to remove the virus inoculum and fresh compounds were added to the infected cells in growth medium (without either DMSO or PEG8000) and incubated at 37°C. Culture medium was replenished after three days with fresh compound. For another set of wells similarly infected with HBV, compounds were added only 72 hours after infection. Seven days following the initial treatment with the SRI-32007 or Myrcludex B, the cell culture supernatants were subjected to HBe antigen ELISA (Cat#WB2496, Xpressbio, Frederick, MD), using the kit protocol with slight modification as described previously for HBV genotype B assay in huh7 cells.

### 2.5. Construction of Plasmid with HBV Core Promoter Fused to Luciferase

DNA plasmid containing HBV core promoter and enhancer 2 (EN2) region was prepared as described previously [10]. A region spanning HBV genome from base pair (bp) 1400 to 1902 was amplified from the cell culture supernatants of HepG2.2.2.15 cells by polymerase chain reaction (PCR) using sense primer 5′-ATCG*GAGCTC*TGGATCCTGCGCGGGAC-3′ and antisense primer 5′-ATCG*AAGCTT*GCCCCAAAGCCA-CCCAAG-3′. The resultant PCR product was digested with Sac I and Hind III restriction enzymes (restriction sites italicized and underlined in the primers) followed by cloning in pGL4.19 (luc2CP/Neo) (Promega, Madison, WI) vector. This vector contained the destabilized firefly luciferase with a short half-life that responds quickly with robust magnitude to changes in transcriptional activity. The cloning facilitated the expression of firefly luciferase gene under the control of HBV core promoter. The resulting plasmid was designated pGL4.19-HBVcore-Luc.

### 2.6. HBV Core Promoter Assay

Huh7 cells were seeded at 2.1 × 10^6^ cells/flask in T75 cell culture flasks using DMEM supplemented with 10% FBS, 380 *μ*g/mL G418, 2.0 mM L glutamine, 100 units/mL penicillin, 100 *μ*g/mL streptomycin, and 0.1 mM nonessential amino acids. Approximately 18–24 hours later, cells were transfected with DNA mixture containing pGL4.19-HBVcore-Luc and a plasmid containing renilla luciferase under the control of SV40 promoter (pRL-SV40, Promega, Madison, WI) using Lipofectamine 2000 reagent (ThermoFisher, Waltham, MA). Four to six hours after transfection, cells were washed with phosphate buffered saline (PBS), trypsinized, and seeded at 2 × 10^4^ cells/well into 96-well plates in a medium containing six different concentrations of the test compounds (in triplicate) and incubated at 37°C incubator containing 5% CO_2_. Seventy-two hours after transfection, cells were harvested by Dual-Glo Luciferase (Promega) reagent according to the manufacturer's protocol to measure the levels of firefly (FLuc) and renilla luciferase (RLuc). Ratio of Fluc/Rluc was normalized to the untreated controls. A parallel set of plates was used for measuring the viability by MTS reagent as described previously.

## 3. Results

### 3.1. Antiviral Activity of SRI-32007 against HBV Genotypes B and D

**Figure 1:**
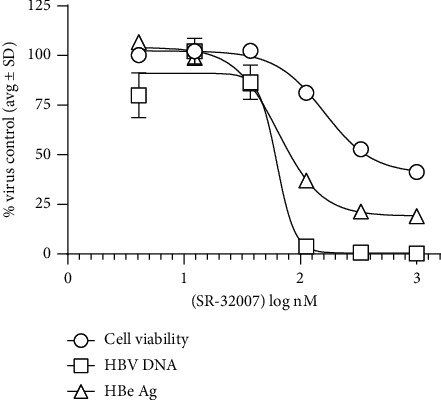
Antiviral activity of SRI-32007 against HBV genotype D in HepG2.2.2.15 cells. HepG2.2.2.15 cells in 96 wells were treated with different concentrations of SRI-32007 for six days followed by measurement of virus associated DNA in the culture supernatants by HBV specific qPCR. HBV DNA and HBe Ag levels in the treated samples and cell viability normalized to the untreated samples (average ± standard deviation from triplicate samples) are shown in the figure.

To identify new chemical entity with antiviral activity, a total of 100 compounds with previous history of clinical evaluation were tested against 12 different viruses, through NIAID antiviral screening program. Out of 100 compounds, SRI-32007 demonstrated anti-HBV activity against HBV genotype D in the HBV virus yield assay. In this assay, HepG2.2.2.15 cells, which constitutively produce HBV (genotype D), were treated with different concentrations of SRI-32007 ranging from 1000 nM to 3 nM in half-log dilutions. After six days of treatment, cell culture supernatants were subjected to HBV specific qPCR to quantitate the HBV associated DNA and HBe Ag ELISA to quantitate HBe Ag levels. Lamivudine was used as a positive control in this assay. Cell viability was measured by MTS assay. HBV DNA, HBe Ag levels, and cell viability of the untreated HepG2.2.2.15 cells were used to normalize the levels in the SRI-32007 or lamivudine treated samples. As shown in Figure 1, SRI-32007 inhibited HBV associated DNA in a dose-dependent manner with cytotoxicity at 1 *μ*M concentration. SRI-32007 demonstrated potent activity against HBV with an EC_50_ of 60.1 nM in the virus yield assay and a CC_50_ of 433 nM (MTS assay) resulting in an SI of 7.2 (Figure 1 and Table 1). Lamivudine (3TC) used in the same assay as a positive control showed an EC_50_ of 20 nM and CC_50_ of >2000 nM (Table 1), with an SI of >100. SRI-32007 reduced HBe Ag levels in the cell culture supernatants, with an EC_50_ of 80 nM and an SI of 5.4. Lamivudine failed to show any effect on HBe antigen levels in HepG2.2.2.15 cells.

**Figure 2:**
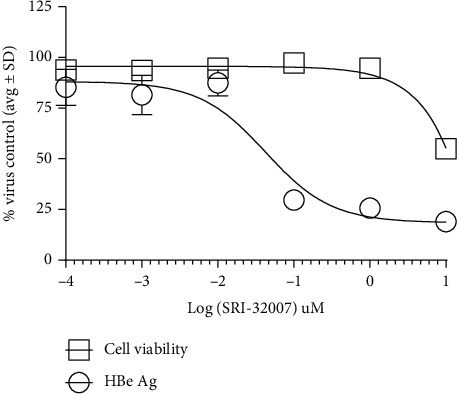
Antiviral activity of SRI-32007 against HBV genotype B in Huh7 cells. Huh7 cells transfected with HBV genotype B plasmid were treated with different concentrations of SRI-32007 in 96 wells for six days followed by measurement of HBe antigen in the culture supernatants by ELISA and cell viability using MTS. HBe Ag levels in the treated samples and cell viability normalized to the untreated samples (average ± standard deviation from triplicate samples) are shown in the figure.

SRI-32007 was also evaluated for antiviral activity against HBV genotype B in huh7 cells. Huh7 cells transfected with plasmid expressing pHBV1.3 B6.2 were treated with serial dilutions of SRI-32007 ranging from 10 *μ*M to 0.0001 *μ*M in triplicate followed by measurement of HBe antigen levels in the cell culture supernatants. As shown in Figure 2, SRI-32007 inhibited HBe antigen levels in the cell culture supernatants in a dose-dependent manner with cytotoxicity at 10 *μ*M concentration. SRI-32007 demonstrated potent activity against HBV genotype B with an EC_50_ of 40 nM in the HBe antigen ELISA assay and a CC_50_ of 10 *μ*M (MTS assay) resulting in an SI of 250. These results demonstrated the antiviral activity of SRI-32007 against HBV genotype D (in HepG2.2.2.15 cells) and genotype B (in huh7 cells).

### 3.2. SRI-32007 Shows Antiviral Activity against HBV Postinfection

Viral entry and transportation into the cells after infection involve microtubules [11]. As the virus yield assay for HBV genotype D in HepG2.2.2.15 cells did not involve the initial infection phase of HBV life cycle, it was not clear whether SRI-32007, a known microtubule inhibitor, would inhibit the HBV infection. To address this question, time of addition (TOA) experiment with SRI-32007 was conducted in HBV infectious assay. This assay involves the infection of HepG_2_ cells constitutively expressing HBV receptor (HepG_2_-NTCP) [9] according to the previously published method. Myrcludex B, a known HBV entry inhibitor [12], was used as a control. HepG_2_-NTCP cells were treated with various concentrations of SRI-32007 ranging from 0.03 *μ*M to 10 *μ*M or Myrcludex B ranging from 0.001 *μ*M to 0.2 *μ*M either at 0.5 hours before or 72 hours after the viral infection. HBe Ag levels in the culture supernatants of HepG2-NTCP cells at seven days postinfection were measured by ELISA assay.

**Figure 3:**
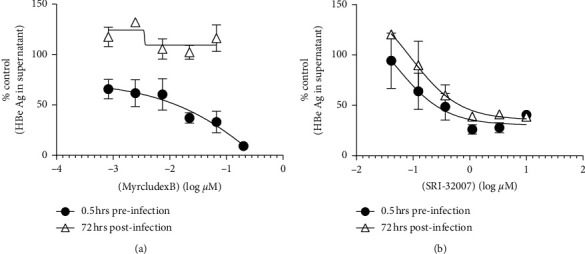
Activity of SRI-32007 in the HBV infectious virus assay in HepG2-NTCP cells. HepG2-NTCP cells were treated with different concentrations of Myrcludex B (a) and SRI-32007 (b) either 0.5 hours before infection or 72 hours postinfection with HBV. HBe Ag levels (mean ± standard deviation) in the culture supernatant as measured by ELISA seven days postinfection normalized to the untreated virus controls are shown in the figure.

Myrcludex B used as a positive control in this experiment showed antiviral activity with an EC_50_ of 12 nM when added at 0.5 hours before infection (Figure 3(a)) similar to the previously published reports [12]. However, when added 72 hours postinfection, Myrcludex B lost its antiviral activity (EC_50_ > 0.2 *μ*M) even at the highest concentration tested (0.2 *μ*M), confirming its antiviral activity through inhibition of HBV entry. Alternatively, SRI-32007 inhibited HBe antigen secretion in the culture supernatant of the infected cells in a dose-dependent manner (Figure 3(b)), whether added 0.5 hours before (EC_50_ of 0.4 *μ*M) or 72 hours after the infection (EC_50_ of 0.6 *μ*M). These results suggested that SRI-32007 exerts its antiviral activity at a post-entry stage either at the viral transcription, replication, or virus yield stage.

### 3.3. SRI-32007 Inhibits HBV Core Promoter Activity

**Figure 4:**
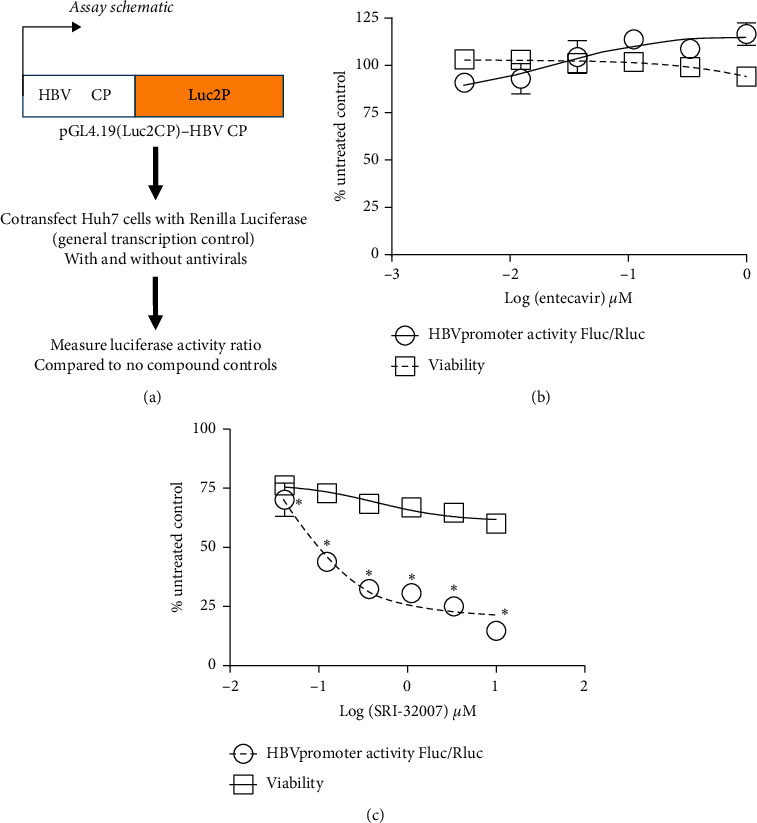
HBV core promoter assay. (a) Schematic of the HBV core promoter assay: (b) and (c) Huh7 cells transfected with plasmid containing HBV core promoter fused to firefly luciferase along with another plasmid encoding renilla luciferase under the control of SV40 promoter were treated with increasing concentrations of SRI-32007 or entecavir, followed by measurement of luciferase activity posttreatment. Ratio of firefly luciferase to the renilla luciferase in Huh7 cells treated with either entecavir (b) or SRI-32007 (c) normalized to the untreated controls (mean ± standard deviation) are shown in the figure. This experiment was repeated twice to confirm the reproducible inhibition of HBV core promoter by SRI-32007. A representative graph from one experiment is shown in the figure. Stars (∗) show statistical significance *p* < 0.05 compared to the untreated control (Dunnett's multiple comparison test, one-way ANOVA, GraphPad Prism 5).

The antiviral activity of SRI-32007 against HBV genotype B (HBe antigen secretion endpoint in huh7 cells), genotype D (virus yield assay endpoint in HepG2.2.2.15 cells), and the HBV infection assay (HepG_2_-NTCP cells with HBe antigen secretion endpoint) suggested that SRI-32007 acts at a post-entry stage. To further determine the anti-HBV MOA, SRI-32007 was evaluated in the HBV core promoter assay (Figure 4(a)). Activity of renilla luciferase was used as an internal control for transfection and transcription in these cells as described previously [13]. Ratio of Fluc and Rluc in this experiment would demonstrate the specific inhibition of HBV core promoter. Entecavir was used as a negative control in this assay to test the specificity of HBV core promoter inhibition by SRI-32007. Entecavir failed to inhibit the HBV core promoter activity (EC_50_ > 1 *μ*M) even at the highest concentration tested in the assay (Figure 4(b)). As entecavir is a HBV polymerase inhibitor [13], lack of inhibition of HBV core promoter dependent luciferase by entecavir suggests the specificity of the HBV core promoter assay. In contrast, in cells treated with SRI-32007, a dose-dependent decrease (inhibition of 29.9 to 85.3% compared to untreated control) in the Fluc/Rluc ratio was observed with an EC_50_ of 0.04 and 0.05 *μ*M and SI of >250 (Figure 4(c)). These results suggest that SRI-32007 inhibits the HBV core promoter.

## 4. Discussion

Current SOC for HBV are able to suppress only viral replication, necessitating the discovery of novel antivirals with the capacity to eliminate the virus. In an effort to identify novel viral inhibitors, compounds with a previous history of clinical experimentations were tested against 12 different viruses. Amongst the compounds tested, SRI-32007 showed antiviral activity against HBV genotypes B and D in two different cell lines: HepG2.2.2.15 and huh7 cells. Reduction in the extracellular virus associated DNA in the presence of SRI-32007 also correlated with the decrease in HBe antigen secreted in the cell culture. The inhibitory effect of SRI-32007 on HBe antigen secretion in cell culture was in contrast to the HBV reverse transcriptase inhibitor lamivudine in this assay system (Table 1). This difference demonstrates the potential disparity in the MOA between SRI-32007 (tubulin inhibitor) and lamivudine (HBV reverse transcriptase inhibitor). Effect on additional viral parameters (decrease in HBe antigen levels as well as HBV virus yield) also suggests the potential benefits conferred by SRI-32007 as an antiviral compared to the HBV polymerase/reverse transcriptase inhibitors.

SRI-32007 (CYT997) is a tubulin polymerization inhibitor with an IC_50_ of 3 *μ*M in the turbidimetric assay [6]. In preclinical animal models, SRI-32007 has shown oral bioavailability in rats (absolute oral bioavailability of 50–70% and a half-life of 2.5 hours) and efficacy in murine cancer models (10 mg/kg/t.i.d.) [6]. In phase I clinical trials, doses of SRI-32007 (CYT997) up to 202 mg/m^−2^ were well-tolerated with pharmacodynamics evidence of efficacy against cancer [14]. Due to these favorable pharmacokinetic (PK) properties previously documented for SRI-32007, its antiviral MOA was further investigated in additional assays for HBV.

SRI-32007 was evaluated in time of addition experiments using HBV infectious assay (HepG_2_-NTCP cells). We anticipated that if SRI-32007 inhibited the HBV infection (entry) stage, then its antiviral activity would be lost upon addition after the infection phase (72 hours post-infection). Alternatively, antiviral activity of SRI-32007 would be still retained, if it inhibited the viral replication/transcription phase. SRI-32007 demonstrated anti-HBV activity in a dose-dependent manner irrespective of the time of addition (i.e., either 0.5 hours before or 72 hours after infection) in the HBV infectious assay. As SRI-32007 retained its activity even when added 72 hours post-infection, SRI-32007 most likely affected the post-infection stage (viral transcription, replication, or virus yield stage) but not early entry events in the HBV life cycle. Effect of SRI-32007 on HBV core promoter activity was further confirmed in the HBV core promoter assay. Huh7 cells were chosen for the HBV core promoter assay due to the robust assay signal shown in the previous reports [13]. Dose-dependent and reproducible inhibition of HBV core promoter suggested that SRI-32007 exerts its anti-HBV activity through inhibition of HBV core promoter. Inhibition of HBV core promoter by SRI-32007 is in agreement with a previous report [15] demonstrating similar effect by several tubulin inhibitors (KX2-391, vincristine, vinblastine, and nocadozole). This report demonstrated the inhibitory effect of tubulin inhibitors specifically on HBV precore promoter activity through HNF4*α* transcription factor inhibition, without affecting the HBV-S1, HBV-S2, or cytomegalovirus promoters. As SRI-32007 belongs to the same class of tubulin inhibitors as mentioned in the work published by Harada et al. [15], SRI-32007 might also possibly exert its anti-HBV activity through the same pathway (i.e., HNF4*α* inhibition).

Tubulin is shown to be important in multiple stages of the virus life cycle for several viruses, such as flu [16], dengue [17], HCV [18], and VSV [19]. These include (1) transportation of virus particles after infection (entry phase), (2) assembly of replication complex during replication, and (3) secretion of virus after replication. Microtubules are also shown to be important at the early events of HBV life cycle in transportation of intracytoplasmic HBV capsid to the nucleus [11] or at latter stages of HBV capsid formation during HBV replication [20]. Inhibition of HBV core promoter by SRI-32007, a tubulin inhibitor, further confirms the importance of microtubules in the post-infection stages of HBV life cycle.

As tubulins are also important for physiological processes of the host cell, the effect of SRI-32007 on cell viability was also evaluated in our experiments. Reduced proliferation/viability of subconfluent cell cultures in the presence of high (micromolar) concentrations of SRI-32007 was observed in the *in vitro* experiments (Figures 1 and 2), similar to KX2-391 [15]. In rats, oral dosing with 25 mg/kg of SRI-32007 results in less than 1 *μ*M plasma concentrations after six hours [6], which correlates with a 0.4 to 0.5 *μ*M concentration range needed to suppress 50% HBV in the HBV infectious assay in our experiments (Figure 3(b)). However, the dose-limiting toxicity (cachexia) (25 mg/kg) observed in mice [6] suggests that achieving effective plasma concentrations of SRI-32007 without adverse events for prolonged time could be challenging. Modest activity (sub-micro molar range) of SRI-32007 in the HBV infectious assay together with anti-proliferative activity observed in the *in vitro* assays might limit the additional preclinical evaluation of SRI-32007 in animal models.

This study demonstrates anti-HBV activity of a repurposed compound SRI-32007 through inhibition of HBV core promoter activity. In addition, our experiments also illustrate the utility of repurposing strategy to identify novel antiviral chemical leads. Further evaluation of structural analogs of SRI-32007, in both the cell-based assays and HBV mouse models, might reveal compounds with better selectivity index against HBV. The discovery and development of selective HBV core promoter inhibitors could be beneficial in identifying novel treatment options for persistent HBV infections.

## Figures and Tables

**Table 1:** Activity of SRI-32007 in the HBV virus yield assay in HepG2.2.2.15 cells.

Compound	HBV virus yield assay			HBe Ag in culture supernatant		
	EC_50_ (nM)	CC_50_ (nM)	Selectivity index (CC_50_/EC_50_)	EC_50_ (nM)	CC_50_ (nM)	Selectivity index (CC_50_/EC_50_)
SRI-32007	60.1	433	7.2	80	433	5.4
Lamivudine/3 TC	20	>2000	>100	>2000	>2000	>1

## Data Availability

The data used to support the findings of this study are available from the corresponding author upon request.
